# Geographical Authentication of *Aquilaria sinensis* Using Integrated C and O Stable Isotope Analysis Coupled with Chemometric Profiling

**DOI:** 10.3390/molecules31071135

**Published:** 2026-03-30

**Authors:** Lei Zeng, Guanghui Lin, Xin He, Jian Qiu, Yoon Soo Kim, Di Liang, Jialin Wei, Minh Mẫn Mai, Jingran Gao

**Affiliations:** 1 The Key Laboratory of Forest Resources Conservation and Utilization in the Southwest Mountains of China Ministry of Education, Southwest Forestry University, Kunming 650224, China; layzeng@139.com (L.Z.); hexin@swfu.edu.cn (X.H.); qiujian@swfu.edu.cn (J.Q.); 19983062864@163.com (J.W.); xn29847435zipa@163.com (M.M.M.); 2 Center for Earth System Science and Institute for Global Change, Tsinghua University, Beijing 100084, China; lingh@tsinghua.edu.cn; 3 Department of Wood Science & Engineering, Chonnam National University, Gwangju 61186, Republic of Korea; kimys@jnu.ac.kr; 4 Xishuangbanna Customs, Kunming Customs District, Xishuangbanna 666100, China; 13708815668@163.com

**Keywords:** wood origin traceability, chemometrics, stable isotope, *Aquilaria sinensis*

## Abstract

Multivariate carbon and oxygen stable isotope analyses combined with chemometric methods were employed to investigate *Aquilaria sinensis* samples collected from six major regions in China (Honghe Hani and Yi Autonomous Prefecture and Xishuangbanna Dai Autonomous Prefecture in Yunnan Province; Zhongshan City and Maoming City in Guangdong Province; and Danzhou City and Chengmai County in Hainan Province). Isotopic δ-values were analyzed across different wood parts (longitudinal and north–south orientations), chemical fractions (de-extracted wood and α-cellulose), and geographical origins. Principal Component Analysis (PCA), Linear Discriminant Analysis (LDA), Support Vector Machine (SVM), Decision Tree, and Random Forest were applied to screen and classify the samples. Four discriminant models were successfully established, achieving a maximum accuracy of 85.7% for distinguishing *Aquilaria sinensis* from different regions, and 88.1% for discrimination at the provincial level. These results demonstrate that stable isotope signatures, when combined with chemometrics, provide a reliable technical approach for the traceability of incense wood and offer a reference framework for verifying the authenticity of Agarwood and related plant-derived materials.

## 1. Introduction

With the rapid growth of forest product trade and the presence of substantial economic interest chains [1], China, one of the world’s largest forest product traders [2], faces an urgent need to objectively assess the impact of timber traceability standards and to establish a comprehensive traceability system. At present, tracing the geographical origin of timber encounters several technical challenges. First, the timber supply chain covers multiple stages, including harvesting, transportation, processing, and sales, during which legality documentation (e.g., logging permits, transport certificates) may be missing or falsified, resulting in incomplete or distorted data chains [2,3]. Second, consumers generally lack specialized knowledge and convenient verification tools, which makes it difficult to independently verify the origins of timber through existing mechanisms such as QR codes or anti-counterfeiting labels. Third, although scientific techniques such as genetic, chemical, and anatomical methods in principle can be used to determine species and geographic origin, the current availability of reference data for geographic origin is limited to a small proportion of priority timber taxa, indicating that only a limited set of tests are currently available for timber provenance verification and that more research and data are needed for broader implementation [1].

Existing timber traceability approaches include blockchain-based systems, mineral element analysis, DNA techniques, and stable isotope analysis. Among these methods, stable isotope analysis has demonstrated broad potential for determining the geographical origins of various products, particularly agricultural commodities and traditional Chinese medicinal materials [4,5,6]. When integrated with other analytical methods, stable isotope techniques have been successfully applied to the traceability of foods and medicinal materials such as milk, tea, and *Cordyceps sinensis* [7,8,9,10,11]. Plant stable isotopes are strongly influenced by environmental factors including temperature, humidity, precipitation, and local isotope compositions [12,13]. For example, carbon isotopes in most plants primarily reflect CO_2_ fixation pathways during photosynthesis, while oxygen isotopes mainly originate from rainfall. Climate transitions (e.g., warm to cool), increases in altitude, or shifts from marine to inland hydrological conditions can alter isotope fractionation, thereby changing relative isotope compositions [14]. As a result, plant stable isotope δ-values vary significantly across different geographical regions. The isotope ratio of a sample:

(1) $$\delta \;=\;(\frac{\text{R-sample}}{\text{R-standard}}\;-\;1)\;\times \;1000‰$$

R-sample represents the isotope abundance ratio of heavy nuclides to light nuclides in the sample; R-standard represents the isotope abundance ratio of internationally recognized reference materials. This principle also applies to wood. Currently, researchers have traced the origin of sandalwood timber by combining stable isotope and mineral element methods [15], while others have provided preliminary explanations for the variation patterns of stable isotope δ values across different wood sections and tree species [16].

Agarwood is produced when *Aquilaria sinensis* trees undergo physical damage (e.g., insect infestation, lightning strikes, animal gnawing, or human drilling), microbial infection (caused by specific fungi or bacteria), or environmental stressors such as drought, high temperature, or chemical induction. These factors activate self-defense mechanisms in the tree, leading to resin oxidation and transformation that ultimately yield agarwood characterized by a distinctive fragrance and pharmacological activities, including sedative, anxiolytic, and anticancer effects [17,18,19]. The two main classes of compounds in *Aquilaria sinensis* resin, namely sesquiterpenes and aromatic derivatives, contribute to its woody, sweet, and floral aroma, thereby supporting its high value in incense, perfumes, and traditional medicine [20,21,22,23]. The primary production regions of *Aquilaria sinensis* in China include Guangdong, Guangxi, Hainan, Fujian, and Yunnan Provinces, with additional distribution across Southeast Asia in countries such as Vietnam, Thailand, and Laos [24]. Variations in ecological environments across these regions lead to differences in the chemical composition of *Aquilaria sinensis*, resulting in notable disparities in agarwood quality and price. For instance, agarwood from Hainan and Vietnam is particularly renowned for its rich and enduring fragrance and typically commands premium market value [25]. With rising consumer interest in health products and traditional culture, global demand for agarwood has been increasing steadily. However, this demand also fuels fraudulent practices in which inferior or counterfeit products are sold as high-grade agarwood for economic gain [26]. Hence, the accurate determination of agarwood authenticity and origin is of critical importance for consumer protection and market regulation.

In this study, we analyze δ-values (δ^13^C and δ^18^O) of *Aquilaria sinensis* collected from six production sites across Yunnan, Guangdong, and Hainan Provinces. Because isotope δ-values can vary not only across different tree parts but also among distinct chemical fractions (e.g., de-extracted wood and α-cellulose), both factors were systematically considered. To enhance provenance discrimination, chemometric approaches including Orthogonal Partial Least Squares Discriminant Analysis (OPLS-DA), LDA, SVM, and clustering methods have previously demonstrated satisfactory performance in food and plant traceability studies. More recently, machine learning methods such as Random Forest have been increasingly applied as predictive algorithms due to their capacity to model complex, nonlinear relationships in large datasets [27,28]. Accordingly, in this work we first apply PCA to reduce dimensionality and extract key discriminant features, followed by the construction of classification models using LDA, SVM, decision trees, and random forests. This multivariate strategy enables robust differentiation of *Aquilaria sinensis* samples from different regions and allows comparison of the classification efficiency of various algorithms. Ultimately, the aim is to identify the optimal predictive model for provenance determination of *Aquilaria sinensis* and to provide a reliable technical solution for agarwood traceability.

## 2. Results and Discussion

### 2.1. δ^13^C and δ^18^O Values of Aquilaria sinensis from Different Regions

The δ^13^C and δ^18^O stable isotope ratios of *Aquilaria sinensis* from six production regions, along with the results of Duncan’s post-hoc variance analysis, are presented in Table 1 and Table 2, while the detailed datasets and additional sampling information are provided in Table S1 (Supplementary Materials). Due to differences in geographical location, soil type, water sources, and climatic conditions, isotopic fractionation occurs within *Aquilaria sinensis*, resulting in significant variations in δ^13^C and δ^18^O across different origins (*p* < 0.05) [29,30,31,32].

Compared with lignin, α-cellulose possesses a relatively simple and stable molecular structure with a fixed chemical formula [33]. Carbon atoms are located primarily in the carbon rings, whereas oxygen atoms occur mainly in hydroxyl groups and ether bonds. Once fixed in the α-cellulose structure, these atoms undergo minimal exchange with the external environment, thereby preserving their isotopic information [34]. As a result, oxygen isotope ratios (δ^18^O) measured in α-cellulose differed significantly from those measured in extract-free wood powder, with the latter consistently showing higher δ^18^O values.

Although isotope values may vary across different plant tissues [25], in this study no significant differences were observed in δ^13^C or δ^18^O among different parts of the same tree (*p* > 0.05). Regression analysis further indicated that geographic origin accounted for 34.0% of the variation in δ^13^C and 35.7% of the variation in δ^18^O, while chemical components contributed 7.1% of the variation in δ^18^O. This highlights the predominant role of geographic factors in shaping the isotopic composition of *Aquilaria sinensis*.

### 2.2. PCA of Stable Isotopes in Aquilaria sinensis

To obtain an intuitive understanding of isotopic variation among different production regions and to evaluate the contribution of each indicator to origin discrimination, PCA was applied to δ^13^C and δ^18^O values measured in extract-free wood powder and to δ^18^O values measured in α-cellulose.

**Figure 1:**
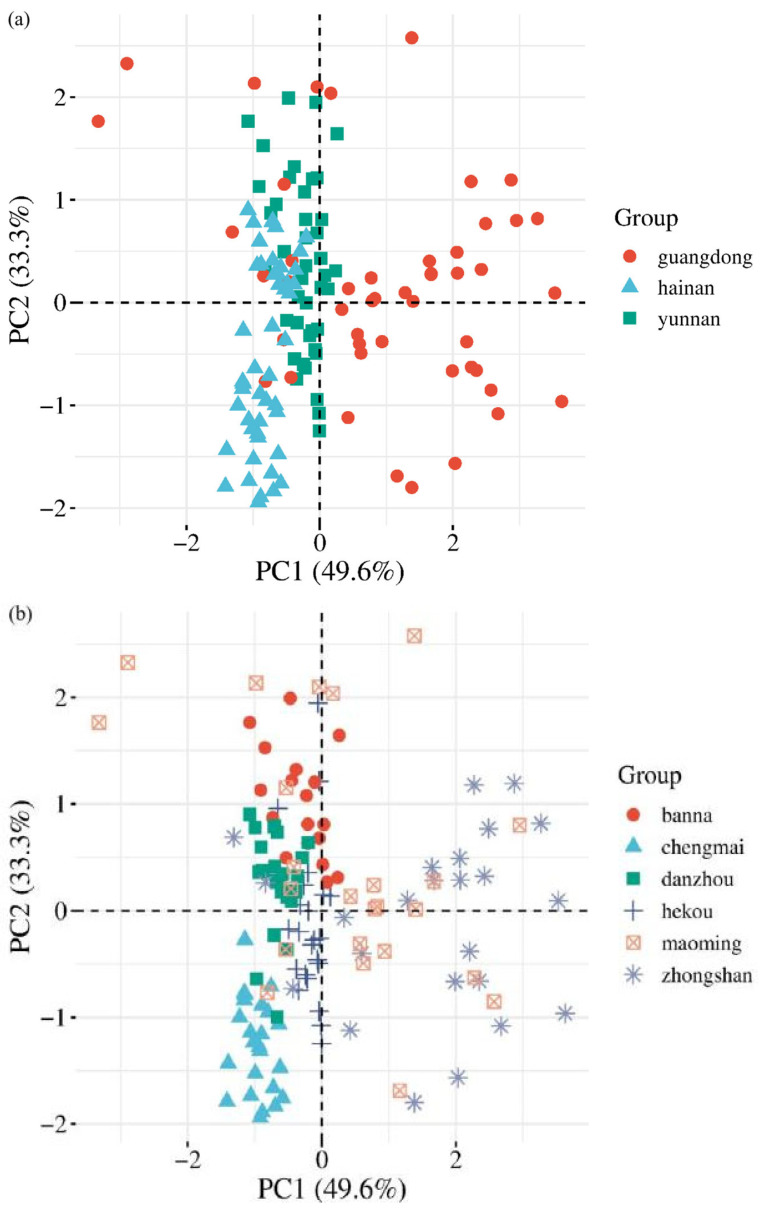
Principal Component Analysis classification results for *Aquilaria sinensis* samples, with accuracy based on province (**a**) and accuracy based on origin (**b**).

The first two principal components (PC1 and PC2) explained 61.27% and 34.31% of the total variance, respectively (Figure 1). For PC1, the weight coefficients were 0.71 for δ^13^C (extract-free), –0.69 for δ^18^O (extract-free), and 0.13 for δ^18^O (α-cellulose). For PC2, the corresponding coefficients were 0.06, 0.25, and 0.97, respectively. The scatterplots (Figure 1a) show that most samples can be distinguished at the provincial level (Yunnan, Hainan, Guangdong), whereas separation at the finer origin level is more difficult, with overlapping distributions across some sites (Figure 1b).

### 2.3. Discrimination Analysis of Stable Isotopes in Aquilaria sinensis

To further explore the feasibility of isotope-based origin tracing, multiple supervised learning approaches were applied, including LDA, Decision Trees, SVM, and Random Forests. These models were trained and validated using δ^13^C and δ^18^O values from extract-free wood powder, along with δ^18^O values from α-cellulose.

#### 2.3.1. LDA

**Figure 2:**
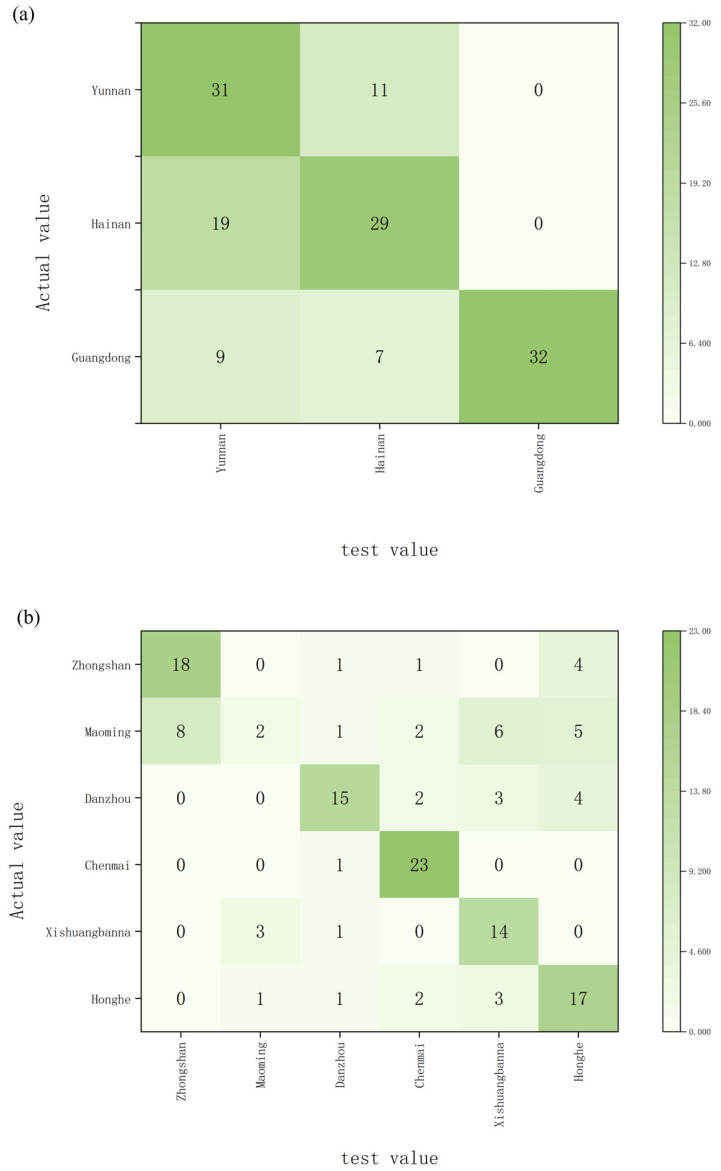
Linear Discriminant Classification results for *Aquilaria sinensis* samples, with accuracy based on province (**a**) and accuracy based on origin (**b**).

Based on the measured δ^13^C and δ^18^O values, a discriminant model for *Aquilaria sinensis* was established using LDA, which is a classical supervised learning method for classification and dimensionality reduction. This projects the data onto a new low-dimensional space where samples from different categories are maximally separated. With priors set to None and projection to two dimensions (n_components = 2), the model was pre-trained using singular value decomposition (SVD). Carbon and oxygen isotopes measured in the extracted wood powder, along with oxygen isotopes measured in α-cellulose, served as indicator pairs. Results shown in Figure 2 indicate that when classifying by province, 66.7% of samples were correctly classified (Figure 2a). When using origin as the classification criterion, 64.5% of samples were correctly classified (Figure 2b). Misclassification primarily occurred between neighboring regions, such as Yunnan and Hainan, suggesting overlapping isotopic signatures across these provinces.

#### 2.3.2. Decision Tree

**Figure 3:**
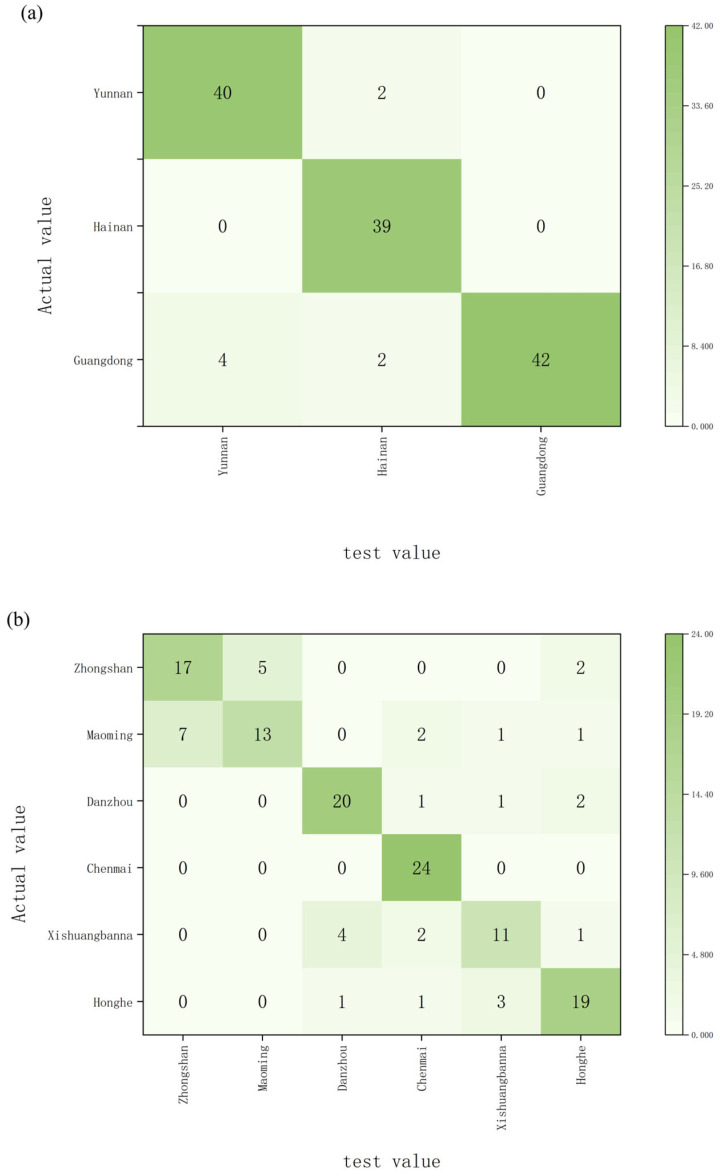
Decision Tree classification results for *Aquilaria sinensis* samples, with accuracy based on province (**a**) and accuracy based on origin (**b**).

Linear discriminant analysis results lack explicit rule-based interpretations, whereas decision trees provide more detailed discrimination criteria. Decision trees are a decision analysis method that evaluates project risks and assesses feasibility by constructing a decision tree based on known probability distributions of various scenarios, thereby determining the probability that the expected net present value is greater than or equal to zero. The decision tree was grown using the Chi-square Automatic Interaction Detection (CHAID) method with a maximum tree depth of 3 (max_depth = 3), a minimum number of cases in parent nodes of 10 (min_samples_split = 10), and a minimum number of cases in leaf nodes of 5 (min_samples_leaf = 5). Carbon and oxygen isotopes measured in wood powder extracts, along with oxygen isotopes measured in α-cellulose, were used as indicators for model prediction. Results indicate that when classifying by province, 87.7% of samples were correctly categorized. Among these, 2 Yunnan samples were misclassified as Hainan, 9 Hainan samples were misclassified as Yunnan, 4 Guangdong samples were misclassified as Yunnan, and 2 Guangdong samples were misclassified as Hainan (Figure 3a). When classifying by origin, 75.4% of samples were correctly categorized (Figure 3b).

#### 2.3.3. SVM

Decision tree analysis provided detailed classification rules; however, its overall predictive performance was limited. Therefore, Support Vector Machine was employed to further improve classification accuracy. SVM is a supervised learning algorithm widely used for binary and multiclass classification tasks. A linear kernel function was adopted, with the penalty parameter C set to 1. Multiclass classification was implemented using a one-versus-rest strategy. Model robustness was evaluated through repeated cross-validation with 10 iterations. Seventy percent of the samples were randomly assigned to the training set, and the remaining thirty percent were used for independent validation.

**Figure 4:**
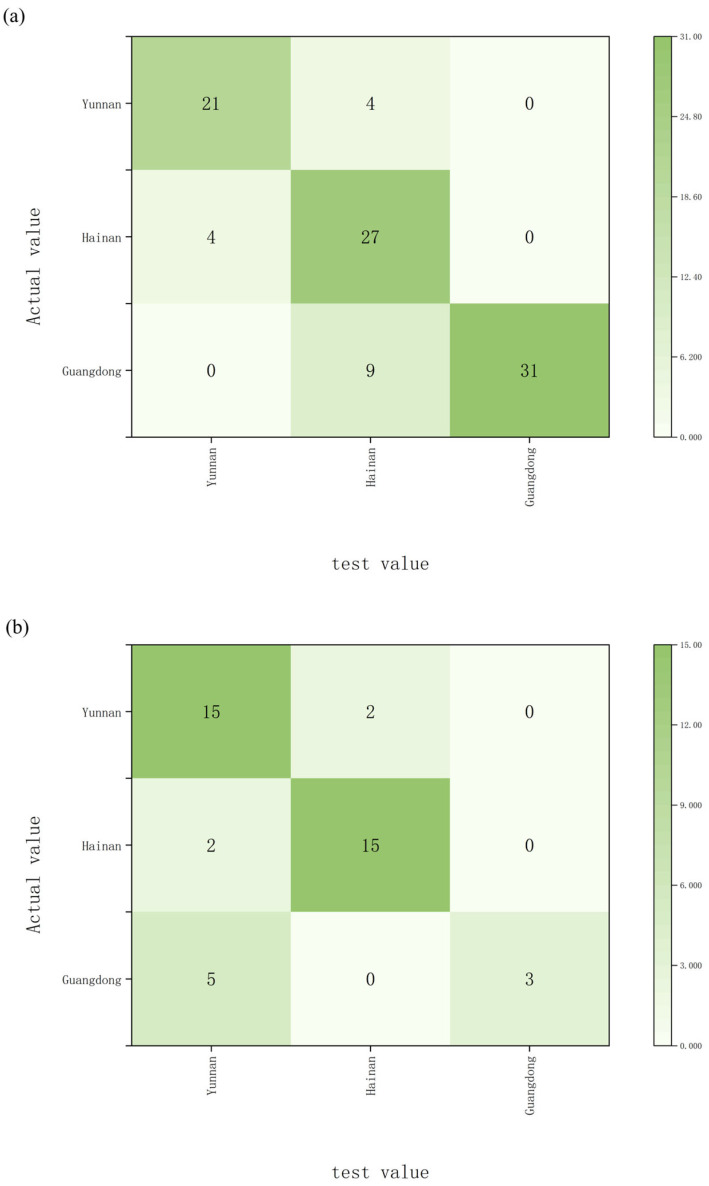

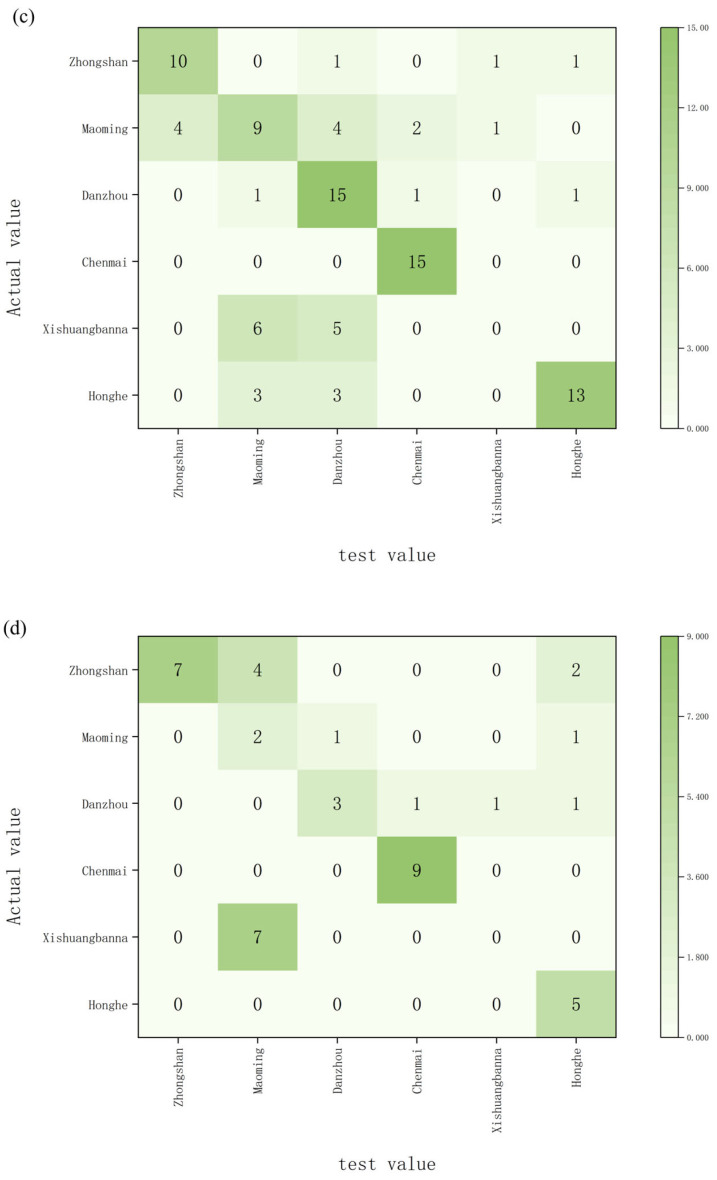
SVM classification results for *Aquilaria sinensis* samples, including: (**a**) training set results with provincial-level accuracy, (**b**) test set results with provincial-level accuracy, (**c**) training set results with origin-level accuracy, (**d**) test set results with origin-level accuracy.

Carbon and oxygen isotope ratios measured in de-extracted wood powder, together with oxygen isotope ratios measured in α-cellulose, were used as predictor variables. When samples were classified at the provincial level, the SVM model achieved a training accuracy of 82.3% and a validation accuracy of 78.6% (Figure 4a,b). For classification by specific origin, the training and validation accuracies were 72.9% and 69.0%, respectively (Figure 4c,d).

#### 2.3.4. Random Forests

Although Support Vector Machine improved classification performance, its interpretability remained limited. A Random Forest model was therefore constructed to enhance predictive accuracy and model robustness. Random Forest is an ensemble learning algorithm that integrates multiple decision trees to reduce variance and improve generalization ability.

The model consisted of 500 decision trees and used Gini impurity as the splitting criterion. Trees were grown without depth restriction, and feature selection at each split was determined automatically. Model stability was assessed using 10-fold cross-validation. Seventy percent of the samples were randomly allocated to the training set, and the remaining thirty percent were reserved for validation.

**Figure 5:**
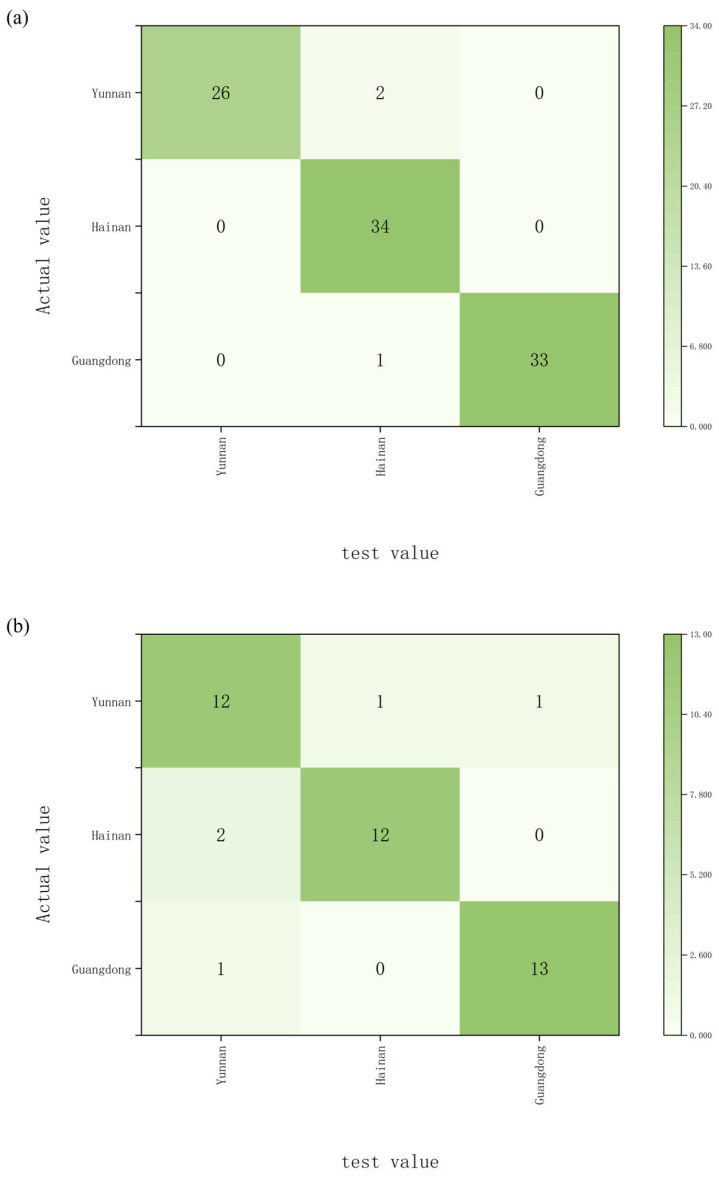

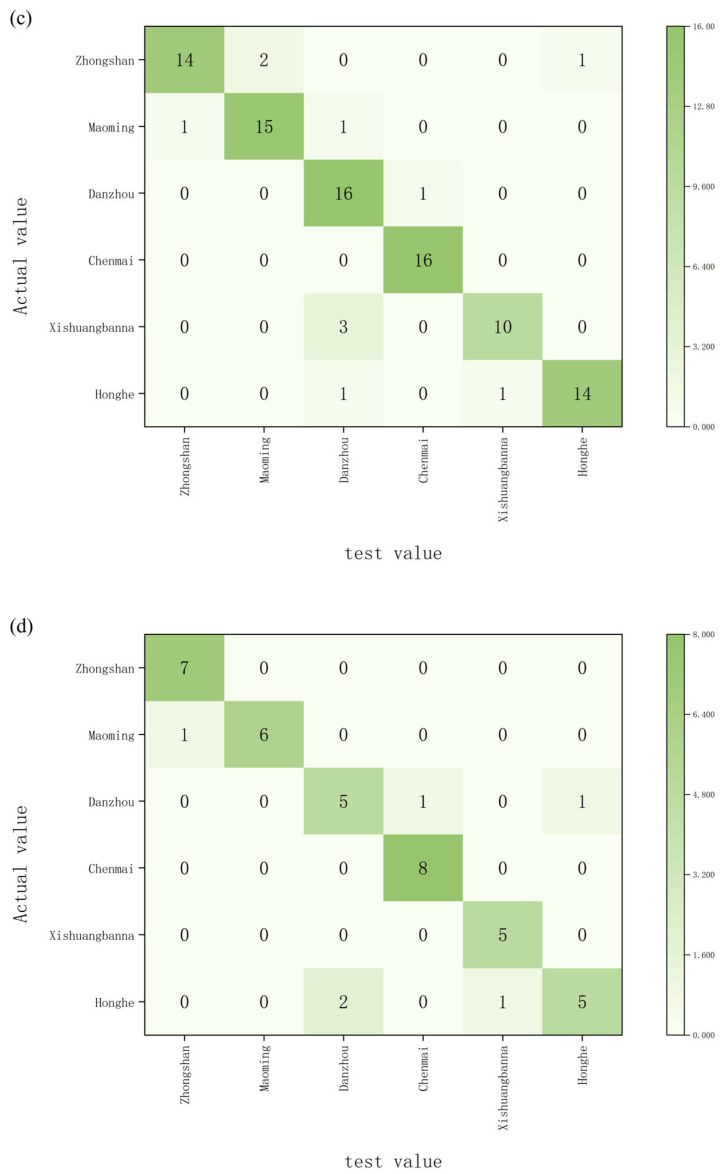
Random Forest classification results for *Aquilaria sinensis* samples, including: (**a**) training set results with provincial-level accuracy, (**b**) test set results with provincial-level accuracy, (**c**) training set results with origin-level accuracy, (**d**) test set results with origin-level accuracy.

Carbon and oxygen isotope ratios measured in de-extracted wood powder, together with oxygen isotope ratios measured in α cellulose, were used as predictor variables. At the provincial classification level, the Random Forest model achieved a training accuracy of 96.9% and a validation accuracy of 88.1% (Figure 5a,b). For classification by specific origin, the training and validation accuracies were 88.5% and 85.7%, respectively (Figure 5c,d).

### 2.4. Discussion

The present study demonstrates that stable carbon and oxygen isotopes provide meaningful geochemical fingerprints for the provenance discrimination of *Aquilaria sinensis*. Significant inter-regional differences in δ^13^C and δ^18^O values (*p* < 0.05) reflect the combined influence of climatic conditions, water sources, altitude, and regional ecohydrological processes [35,36,37,38]. δ^13^C values are primarily governed by photosynthetic discrimination and water use efficiency, which are sensitive to precipitation regimes and atmospheric humidity. Meanwhile, δ^18^O values largely reflect the isotopic composition of source water and evaporative enrichment during transpiration. The regression analysis further confirmed that geographic origin explained over one third of the isotopic variance (34.0% for δ^13^C and 35.7% for δ^18^O), highlighting the dominant role of environmental controls in shaping isotopic signatures.

Notably, δ^18^O values measured in α-cellulose differed systematically from those measured in extract-free wood powder. This difference mainly reflects the more homogeneous chemical composition of α-cellulose compared with bulk wood components. α-cellulose consists of a structurally uniform polysaccharide matrix in which oxygen atoms are primarily bound within hydroxyl and ether functional groups. Because of this clearly defined structure, the oxygen isotope composition of α-cellulose more directly reflects the isotopic signature of source water and leaf water enrichment during cellulose formation [38,39]. In contrast, extract-free wood powder contains a mixture of structural polymers, including cellulose, hemicellulose, and lignin, each of which may exhibit slightly different isotopic signatures [40]. The presence of these heterogeneous components can introduce additional isotopic variability and partially obscure the environmental signal recorded in cellulose. The observed 7.1% contribution of chemical components to δ^18^O variation confirms that biochemical fractionation exists but plays a secondary role relative to geographic influences. This finding supports the use of α-cellulose as a more reliable proxy in oxygen isotope-based provenance studies.

PCA results indicated that isotopic variables alone provide partial but not complete separation among origins. The first two principal components explained more than 95% of total variance, indicating that most of the isotopic variability in the dataset is captured by these components. However, overlap among neighboring regions reflects shared climatic gradients and similar eco environmental conditions. This demonstrates that while stable isotopes contain strong environmental signals, linear dimensionality reduction alone is insufficient for high-resolution discrimination.

The comparative evaluation of supervised learning models further clarifies the discriminatory power of isotopic indicators. LDA, as a linear classifier, achieved moderate accuracy, indicating that class boundaries are not strictly linear. Decision trees improved interpretability but remained limited by their tendency to over-partition data. SVM enhanced generalization capacity through margin maximization, yielding improved classification accuracy. However, Random Forests achieved the highest performance, with provincial-level test accuracy of 88.1% and origin-level test accuracy of 85.7%.

Random Forest achieved the best overall classification performance among the tested models. One important reason is that Random Forest is an ensemble learning algorithm that combines the predictions of many decision trees, which improves model stability and reduces the risk of overfitting compared with individual decision tree classifiers. This property is particularly advantageous when dealing with datasets that have complex structures or partially overlapping distributions. In the present study, the isotopic signatures (δ^13^C and δ^18^O) of *Aquilaria sinensis* showed a certain degree of overlap among different geographical regions, especially at finer spatial scales. Such overlap may reduce the effectiveness of linear classifiers such as Linear Discriminant Analysis and may also limit the performance of Support Vector Machines when simple kernel functions are applied. In contrast, Random Forest can capture nonlinear relationships and interactions among variables without requiring strict assumptions about data distribution. In addition, the use of bootstrap sampling and random feature selection during model construction increases model robustness and improves predictive performance when the number of predictor variables is relatively small. Because the dataset in this study included only three isotopic indicators but exhibited clear regional variability, the ensemble structure of Random Forest was better able to identify subtle multivariate patterns in the isotopic data. Similar advantages of Random Forest have been reported in other authenticity and provenance studies using isotopic or elemental datasets, where Random Forest often outperforms traditional linear classification methods such as LDA when applied to complex environmental datasets [41,42,43]. These characteristics likely contributed to the superior discrimination accuracy achieved by the Random Forest model in both provincial-level and origin-level classifications in this study.

Combined isotope and elemental analyses have been shown to improve discrimination performance in geographic origin studies, but such approaches typically require additional analytical instrumentation such as ICP MS in addition to isotope ratio mass spectrometry and involve more complex laboratory procedures, which can substantially increase operational costs and analytical time, as reported in previous provenance and authenticity studies combining elemental and isotopic fingerprints [44]. In contrast, the present study demonstrates that reliable provenance discrimination can be achieved using a limited set of isotopic indicators. Classification based solely on three isotopic variables, namely δ^13^C and δ^18^O measured in extract-free wood powder and δ^18^O measured in α-cellulose, combined with Random Forest modeling, yielded classification accuracies exceeding 85%. This level of performance is comparable to results reported in other authenticity and provenance studies using stable isotope data, where accuracies in the range of approximately 80–90% are commonly observed [45,46,47]. These findings suggest that stable isotope analysis alone can provide a practical and technically feasible approach for geographic origin verification, particularly in situations where rapid screening and cost efficiency are important considerations. Consequently, stable isotope-based methods represent a promising tool for the provenance authentication of agarwood and other high-value timber resources.

Nevertheless, certain limitations remain. The study was restricted to six sampling regions, and isotopic signatures may vary temporally in response to climatic variability [35,36]. Expanding the geographic coverage and incorporating multi-year sampling would improve model robustness. Furthermore, integrating additional isotopic systems such as hydrogen or strontium isotopes, or combining isotopic and elemental fingerprints may further enhance discriminatory power in provenance studies [44,48].

Overall, stable isotope analysis combined with ensemble machine learning provides a robust and practical framework for the provenance verification of *Aquilaria sinensis*, offering a simpler analytical strategy than approaches requiring combined elemental and isotopic fingerprints [49].

## 3. Materials and Methods

### 3.1. Sample Collection and Preparation

A total of 138 *Aquilaria sinensis* samples were collected from six production sites across three provinces, derived from 90 individual trees (15 trees per site). To assess the influence of different plant parts on isotopic δ-values (δ^13^C and δ^18^O), three trees from each site were selected for multi-position sampling. For these trees, wood samples were collected at three heights (40 cm, 110 cm, and 180 cm above ground level) on both the north and south sides of the trunk. The remaining 12 trees at each site were sampled only at 40 cm above ground level on the south side.

**Figure 6:**
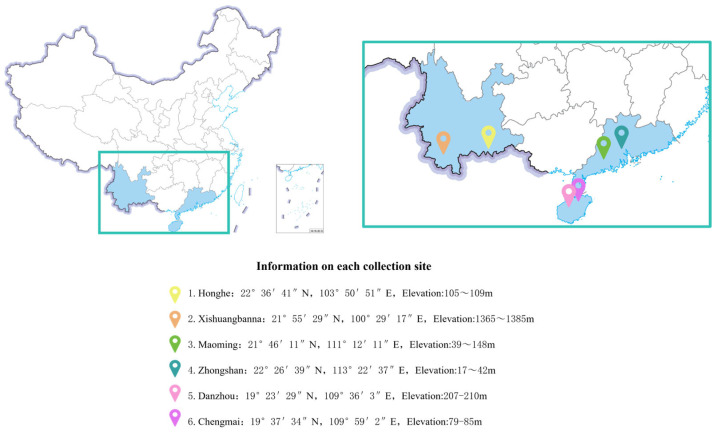
Information on six sampling locations for *Aquilaria sinensis*.

The specific information of the six production areas where samples were collected is as follows: Honghe Hani and Yi Autonomous Prefecture is located at 22°36′41″ N, 103°50′51″ E, altitude about 105~109 m; Xishuangbanna Dai Autonomous Prefecture is located at 21°55′29″ N, 100°29′17″ E, altitude about 1365~1385 m; Maoming City is located at 21°46′11″ N, 111°12′11″ E, altitude about 39~148 m; Zhongshan City is located at 22°26′39″ N, 113°22′37″ E, altitude about 17~42 m; Danzhou City is located at 19°23′29″ N, 109°36′3″ E, altitude about 207~210 m; Chengmai County is located at 19°37′34″ N, 109°59′2″ E, altitude about 79~85 m. The sample distribution was as follows: Honghe Hani Yi Autonomous Prefecture (24 samples), Xishuangbanna Dai Autonomous Prefecture (18 samples; here, due to artificial height control without fertilization, the maximum sampling height did not exceed 180 cm), Maoming City (24 samples), Zhongshan City (24 samples), Danzhou City (24 samples), and Chengmai County (24 samples) (Figure 6). All samples were coded by geographical origin for subsequent analysis.

Collected wood samples were obtained from living *Aquilaria sinensis* trees in the field using a growth increment borer. The initial weight of each collected sample ranged from 8 g to 15 g. After collection, the specimens were oven-dried at 60 °C for 48 h until a constant weight was achieved. The dried wood was then ground using a pulverizer, passed through a 100 mesh sieve with an aperture of 0.15 mm, and thoroughly homogenized [40].

Because the distinction between heartwood and sapwood in *Aquilaria sinensis* is not pronounced, all woody tissues were ground together and mixed thoroughly before subsampling. The prepared samples were subsequently stored in sealed containers until isotope analysis.

### 3.2. Sample Preparation and Stable Isotope Analysis

Wood samples were processed to obtain two chemical fractions for stable isotope analysis: extract-free wood powder and purified α-cellulose. These fractions were analyzed to evaluate potential compositional effects on oxygen isotope measurements. To obtain extract-free wood for stable isotope analysis, approximately 2–3 g of the homogenized wood powder was subjected to solvent extraction following a modified Soxhlet extraction procedure. The samples were first extracted with a mixture of toluene and ethanol (2:1, *v*/*v*) for 6 h to remove lipophilic extractives such as resins, waxes, and fats. Subsequently, the samples were extracted with 95% ethanol for 4 h to remove additional soluble organic compounds, including low-molecular-weight phenolics and pigments. Finally, the samples were rinsed with hot distilled water to remove remaining water-soluble substances such as sugars, inorganic salts, and tannins. After extraction, the residues were oven dried at 60 °C to constant weight and stored in sealed containers until further isotope analysis. This procedure removes non-structural extractives while retaining the structural components of wood, thereby improving the reliability of stable isotope measurements. The extraction protocol followed commonly used procedures in wood chemistry and stable isotope studies [40,50,51]. The α-cellulose fraction was isolated using a widely adopted chlorite extraction procedure for wood cellulose preparation with minor modifications. Wood samples were first subjected to solvent extraction using ethanol benzene or methanol to remove resins and other extractive compounds with low molecular weight. Lignin was subsequently removed by repeated treatments (3–6 cycles) with an acidified sodium chlorite solution (~1% NaClO_2_) at 70 °C until the samples became white. Hemicellulose was then removed using a 17% NaOH solution to obtain purified α-cellulose. The resulting material was thoroughly rinsed with deionized water until neutral pH was reached, followed by filtration, drying, and fine grinding prior to stable isotope analysis. This procedure removes non-cellulose components while preserving the structural cellulose fraction suitable for isotope measurements [50,51,52]. Care was taken to completely eliminate chemical residues to prevent potential isotopic interference.

Stable isotope analyses were conducted using an elemental analyzer coupled to an isotope ratio mass spectrometer (EA-IRMS, Thermo Fisher Scientific, Bremen, Germany). Carbon isotope ratios (δ^13^C) were measured using a DELTA V Advantage Isotope Ratio Mass Spectrometer (Thermo Fisher Scientific, Bremen, Germany) coupled with a Flash 2000 Elemental Analyzer (Thermo Fisher Scientific, Bremen, Germany). Approximately 2–3 mg of extract-free wood powder was weighed into tin capsules and combusted at high temperature in the elemental analyzer. The combustion process converted sample carbon into CO_2_, which was subsequently introduced into the IRMS for determination of the ^13^C/^12^C ratio.

Oxygen isotope ratios (δ^18^O) were determined using the same IRMS system coupled with a high-temperature conversion elemental analyzer (TC/EA, Thermo Fisher Scientific, Bremen, Germany). Approximately 0.2–0.3 mg of extract-free wood powder or purified α-cellulose was sealed in silver capsules and pyrolyzed at high temperature (approximately 1350–1450 °C) to convert oxygen in the samples to CO gas. The resulting CO was introduced into the IRMS for determination of the ^18^O/^16^O ratio.

Isotope ratios are reported in delta (δ) notation relative to international standards, Vienna Pee Dee Belemnite (VPDB) for carbon and Vienna Standard Mean Ocean Water (VSMOW) for oxygen. Instrument calibration and drift correction were performed using internationally recognized reference materials USGS40 and USGS41a (L-glutamic acid), analyzed together with unknown samples in each analytical sequence. A linear normalization procedure based on the certified δ values of these standards was applied to correct for instrumental bias.

Each sample was measured in triplicate, and the reported δ values represent the arithmetic mean of the measurements. Analytical precision, determined from repeated measurements of reference materials, was better than ±0.1‰ for δ^13^C and ±0.3‰ for δ^18^O.

In this study, δ^13^C values were determined from extract-free wood powder, whereas δ^18^O values were measured in both extract-free wood powder and purified α-cellulose to evaluate potential compositional effects.

### 3.3. Statistical Analysis

Data processing and figure generation were performed using Origin 2021 (OriginLab, Northampton, MA, USA) and WPS Office (including WPS Writer and WPS Spreadsheets, version 12.1.0.25225; Kingsoft Office Software Corporation, Beijing, China, 2026). Statistical analyses were conducted using SPSS 27.0 (IBM SPSS, Chicago, IL, USA) and Python (version 3.14).

Prior to statistical analysis, all datasets were tested for normality (Shapiro–Wilk test) and homogeneity of variance (Levene’s test). One-way analysis of variance (ANOVA) was applied to evaluate the influence of different factors on δ^13^C and δ^18^O values when parametric assumptions were satisfied. When necessary, appropriate data transformations were performed. Statistical significance was set at *p* < 0.05.

Linear regression analyses were performed in Python to quantify the relative contributions of individual environmental or compositional factors to isotopic variation. Model performance was evaluated based on the coefficient of determination (R^2^) and root mean square error (RMSE).

Before multivariate analysis, variables were standardized (z-score normalization) to eliminate scale effects. PCA was applied to reduce dimensionality and identify major discriminant variables. LDA was used to evaluate group separation and construct provenance classification functions.

Supervised machine learning models, including SVM, decision trees, and random forests (RF), were established to develop provenance traceability models. Model hyperparameters were optimized using grid search procedures. To avoid overfitting, model performance was assessed using stratified 10-fold cross-validation. Additionally, 70% of the samples were randomly selected as the training dataset, and the remaining 30% were reserved as an independent test set for external validation. Classification performance was evaluated using overall accuracy, precision, recall, and F1-score.

## 4. Conclusions

In summary, this study analyzed δ values (δ^13^C, δ^18^O) of *Aquilaria sinensis* collected from six production regions across Yunnan, Guangdong, and Hainan provinces, considering variations among plant parts and chemical components. Significant differences in isotope compositions were observed across origins. Principal component analysis further confirmed that δ^13^C and δ^18^O provide strong discriminatory power for tracing the geographical provenance of *Aquilaria sinensis*.

Among the tested classification models, Random Forest achieved the highest recognition performance, with the combined use of δ^13^C and δ^18^O values yielding discrimination accuracies of 88.1% at the provincial level and 85.7% at the origin level.

Nevertheless, due to the limited sample size, further expansion, calibration, and validation of the model are necessary to enhance its accuracy, robustness, and practical applicability. Future work may integrate additional isotopic indicators or combine isotopic and elemental analyses to further improve provenance discrimination of *Aquilaria sinensis*.

## Figures and Tables

**Table 1:** Stable Isotope Analysis Results for *Aquilaria sinensis* Across Different Provinces.

Region	δ^13^C‰ (Extract-Free Wood Powder)	δ^18^O ‰ (Extract-Free Wood Powder)	δ^18^O ‰ (α-Cellulose)
Yunnan (*n* = 42)	−26.75 ± 0.78 ^a^ (−27.53~−25.97)	20.76 ± 0.60 ^a^ (20.16~21.36)	21.94 ± 0.80 ^a^ (21.14~22.74)
Hainan (*n* = 48)	−27.55 ± 0.83 ^b^ (−28.38~−26.72)	21.60 ± 0.57 ^c^ (21.03~22.17)	23.02 ± 0.72 ^c^ (22.30~23.74)
Guangdong (*n* = 48)	−26.92 ± 0.94 ^a^ (−27.86~−25.98)	18.35 ± 3.72 ^b^ (14.63~22.07)	19.84 ± 3.15 ^b^ (16.69~22.99)

Note: Values represent means ± standard deviations; at the *p* = 0.05 significance level, different lowercase letters denote significant differences.

**Table 2:** Stable Isotope Analysis Results for *Aquilaria sinensis* from Different Origins.

Region	δ^13^C‰ (Extract-Free Wood Powder)	δ^18^O‰ (Extract-Free Wood Powder)	δ^18^O ‰ (α-Cellulose)
Honghe (*n* = 24)	−27.19 ± 0.67 ^b^ (−27.86~−26.52)	20.66 ± 0.43 ^b^ (20.23~21.09)	21.87 ± 0.67 ^b^ (21.20~22.54)
Xishuangbanna (*n* = 18)	−26.16 ± 0.46 ^d^ (−26.62~−25.70)	20.90 ± 0.43 ^b^ (20.47~21.33)	22.04 ± 0.67 ^b^ (21.37~22.71)
Chenmai (*n* = 24)	−28.26 ± 0.41 ^a^ (−28.67~−27.85)	21.98 ± 0.52 ^c^ (21.46~22.50)	23.17 ± 0.71 ^c^ (22.46~23.88)
Danzhou (*n* = 24)	−26.85 ± 0.42 ^c^ (−27.27~−26.43)	21.22 ± 0.31 ^c^ (20.91~21.53)	22.87 ± 0.70 ^c^ (22.17~23.57)
Zhongshan (*n* = 24)	−27.17 ± 0.75 ^b^ (−27.92~−26.42)	17.28 ± 2.92 ^a^ (14.36~20.20)	18.54 ± 3.22 ^a^ (15.32~21.76)
Maoming (*n* = 24)	−26.68 ± 1.06 ^bcd^ (−27.74~−25.62)	19.41 ± 4.17 ^b^ (15.24~23.58)	21.13 ± 2.53 ^b^ (18.60~23.66)

Note: Values represent means ± standard deviations; at the *p* = 0.05 significance level, different lowercase letters denote significant differences.

## Data Availability

Data will be made available upon request.

## Supplementary Materials

The following supporting information can be downloaded at: https://www.mdpi.com/article/10.3390/molecules31071135/s1, Table S1: Raw stable isotope dataset (δ^13^C and δ^18^O values) and sample information of *Aquilaria sinensis* analyzed in this study.
